# Reactivation of BK Polyomavirus in Urine Cytology Is Not Associated with Urothelial Cell Carcinoma

**DOI:** 10.3390/v12121412

**Published:** 2020-12-08

**Authors:** Faisal Klufah, Ghalib Mobaraki, Axel zur Hausen, Iryna V. Samarska

**Affiliations:** 1 Department of Pathology, GROW—School for Oncology & Developmental Biology, Maastricht University Medical Centre+, 6229 HX Maastricht, The Netherlands; g.mobaraki@maastrichtuniversity.nl (G.M.); axel.zurhausen@mumc.nl (A.z.H.); 2 Department of Laboratory Medicine, Faculty of Applied Medical Sciences, Albaha University, Albaha 65779, Saudi Arabia; 3 Department of Medical Laboratories Technology, Faculty of Applied Medical Sciences, Jazan University, Jazan 45142, Saudi Arabia

**Keywords:** BKPyV, small DNA viruses, bladder cancer, cancer, polyomavirus, tumorigenesis, decoy cells

## Abstract

BK polyomavirus (BKPyV) has been associated with some high-grade and special urothelial cell carcinoma (UCC) subtypes in immunosuppressed patients. Here, we evaluated the relationship of BKPyV-positive urine cytology specimens (UCS) with UCC. A large single-institution database was retrospectively searched for UCS positive for decoy cells, suggesting BKPyV infection. These were tested for the presence of BKPyV by PCR and immunohistochemistry (IHC) in urine sediments and formalin-fixed paraffin-embedded (FFPE) tissue samples of UCC. Decoy cells were reported in 30 patients out of the database with 22.867 UCS. Of these 30 patients, 16 (53.3%) had no history of UCC. Six patients out of these 16 had a history of transplantation, 4 had a history of severe chronic medical conditions, and 6 had no chronic disease. The other fourteen patients were diagnosed with either in situ or invasive UCC of the urinary bladder (14/30; 46.6%) prior to the detection of decoy cells in the urine. Nine of these UCC patients received intravesical treatment (BCG or mitomycin) after the first presentation with UCC. However, the clinical data on the treatment of the other five UCC patients was lacking. IHC identified BKPyV-positivity in the urine samples of non-UCC and UCC patients, while no BKPyV positivity was found in FFPE tissues of primary UCCs and metastases. In addition, BKPyV-PCR results revealed the presence of BKPyV DNA in the urine of the UCC cases, yet none in the UCC tissues itself. These data strongly indicate that BKPyV reactivation is not restricted to immunosuppression. It can be found in UCS of the immunocompetent patients and may be related to the intravesical BCG or mitomycin treatment of the UCC patients.

## 1. Introduction

BK polyomavirus (BKPyV) is a human polyomavirus that has been suspected as a putative oncogenic virus in the development of urothelial cell carcinomas (UCC) in immunocompromised patients [1,2]. Approximately 90% of the human population is infected during early life with BKPyV. The virus remains latent in the epithelium of the proximal tubule of the kidney, within the urothelial cell layer of the bladder, ureters, renal pelvis, and other tissue types [1]. BKPyV reactivation frequently occurs following immunosuppression, especially in the context of kidney and solid organ transplantations, with the risk of developing BKPyV-associated nephropathy (PyVAN) [3,4]. BKPyV replication has been described to be a significant risk factor for bladder cancer development following kidney transplantation [2,5]. Thus, BKPyV-positivity has been reported previously in the urinary bladder and kidney tumors occurring after PyVAN [2,5,6,7,8,9,10]. The spectrum of the BKPyV positive urinary bladder lesions includes high-grade urothelial carcinoma (UCC; both in situ and invasive), micropapillary urothelial carcinoma, pleomorphic giant cell carcinoma, bladder adenocarcinoma, and nephrogenic adenoma [3,5,6,7,8,9]. Moreover, BKPyV-DNA was detected in many other tumors, including pancreas, liver, oral, oropharyngeal, laryngeal squamous cell carcinomas, rhabdomyosarcoma, Kaposi’s sarcoma, prostate adenocarcinoma, and brain tumors [2,10,11]. In 2012, the International Agency for Research on Cancer (IARC) classified BKPyV as a group 2B possibly carcinogenic candidate to humans [2].

BKPyV-infected cells from renal tubules and urothelium can be detected by cytology as decoy cells that have enlarged and altered nuclei with large homogeneous basophilic nuclear inclusions, which mimic cellular changes observed in UCC in situ [3,12]. The identification of BKPyV-IHC positive decoy cells in voided urine has thus been interpreted as a strong indicator of BKPyV reactivation in urothelial cells following immunosuppressive treatment [3,4]. These cells have been shown to express BKPyV large T antigen (LTAg) using immunohistochemistry (IHC) [7]. Due to its known cross-reactivity with the LTAg of BKPyV, an anti-Simian virus 40 (SV40) LTAg antibody is used in clinical practice as a surrogate marker of BKPyV infection [1,13].

The aim of this study was to assess the relation of BKPyV-positive urine cytology specimens (UCS) to the detection of UCC in a large UCS database and the following evaluation of BKPyV in the UCC of the urinary bladder. In addition, we aimed to evaluate the relation of BKPyV to intravesicular BCG or mitomycin treatment of UCC patients.

## 2. Materials and Methods

### 2.1. Study Population

The starting point of this study was a single-institution database that included 22.867 UCS. This database was searched for UCS containing decoy cells as detected by cytology over a 15 year period (January 2004–December 2019) using the laboratory information system of the department of pathology, Maastricht University Medical Center+ (MUMC+), the Netherlands. Thirty patients were identified and included in this study for further workup and analyses. All cytology slides, including immunocytochemistry for SV40, were retrieved and independently reviewed by two pathologists (IVS and AzH). Cases that also had bladder biopsies or resections obtained following and preceding the urinary cytology specimen were collected from the archive and independently reviewed. Clinical information regarding medical history, transplantation, chronic medical condition (autoimmune disease, cancer, diabetes, immunosuppressive treatment, chemotherapy, radiotherapy, and connective tissue disease), as well as the history of renal and bladder tumors were obtained from the medical records of the MUMC+. This study was approved by the Medical Ethics Review Committee of the Maastricht University Medical Center in the Netherlands (2019-0977). All specimens were collected and studied in accordance with the protocol of the Dutch Code of Conduct for Observational Research with Personal Data (2004) and Tissue [14].

### 2.2. Cytology

Unfixed urine samples were prepared by the cytospin method, according to the previously described protocol [15]. One cytospin was stained with Papanicolaou stain, screened by a cytotechnologist, and subsequently reviewed and reported by a pathologist. The other cytospin was stained with the immunoperoxidase staining for SV40 LTAg immunocytochemistry (ICC). The ICC was performed using SV40 LTAg antibody (clone: PAb416, dilution 1:500, Calbiochem Inc., San Diego, CA, USA) with a Dako’s autostainer Link 48 using the EnVision FLEX visualization kit (K8008, DAKO, Carpinteria, CA, USA) according to the diagnostic standard routine and manufacturer’s protocol. Only nuclear staining was regarded as a positive reaction for the SV40 antibody.

### 2.3. Histology

Urinary bladder biopsy and resection specimens were fixed in 4% buffered formalin and embedded in paraffin, according to the routine pathology diagnostic procedures. For light microscopy, hematoxylin and eosin (H&E)–stained sections were performed on 3–5 μm-thick tissue sections.

### 2.4. Immunohistochemistry

Representative formalin-fixed paraffin-embedded tissues (FFPE) were chosen from bladder biopsy and resection cases, and IHC was performed using the SV40 LTAg antibody (clone: PAb416, dilution 1:500, Calbiochem Inc. San Diego, CA, USA). The IHC staining was performed on 3–5 μm-thick FFPE sections using Dako’s autostainer as described above in the cytology section. Only nuclear protein expression was interpreted as a positive reaction for the SV40 antibody. All slides were scanned by the VENTANA iScan-HT slide scanner (Roche Diagnostics Inc., Tucson, AZ, USA).

### 2.5. BKPyV PCR

Nine FFPE specimens and five urine sediments of UCC-patients were retrieved from the archive at the Department of Pathology, Maastricht University Medical Center+. DNA was extracted from all specimens using the protocol of genomic DNA isolation by (NucleoSpin^®^ Tissue, Macherey–Nagel GmbH & Co., Düren, Germany). The DNA concentration was assessed using a spectrophotometer (NanoDrop 2000, Thermo Scientific., Wilmington, DE, USA). Per sample, 125 ng DNA were added to the PCR reaction. All isolated DNAs were assessed for quality and integrity using multiplex primers (SCS: specimen control size) as described previously [16,17].

Two PCR primer sets were used to screen for the presence of BKPyV DNA. One primer set targeted the viral LTAg, while the other targeted the viral protein 1 (VP1), all primer sequences listed in Supplementary Table S3. Sequencing of all obtained PCR products was compared and analyzed with the reference sequences of the National Center for Biotechnology Information (NCBI), Entrez nucleotide database using the NCBI Blast program.

## 3. Results

### 3.1. Study Population and Urine Cytology

**Figure 1:**
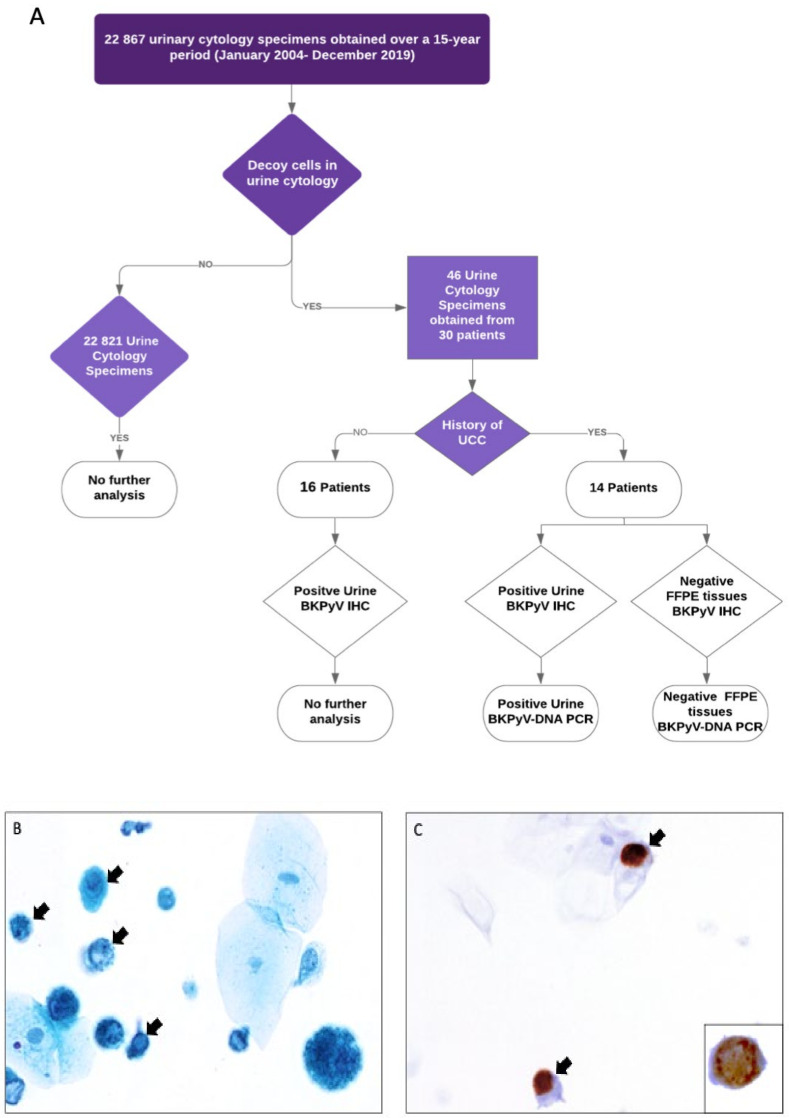
(**A**) Flow chart depicting the study design and immunohistochemistry results. (**B**) Decoy cells (arrows) in urine cytology (Papanicolaou staining) and (**C**) immunohistochemistry for BKPyV (arrows), and an enlarged image in the lower right corner showing the expression of BKPyV. The images were taken at 200× magnification.

Of the 22.867 retrospectively evaluated UCS, 46 urine specimens (0.2%), obtained from 30 patients, were positive for decoy cells, indicating BKPyV-infection (Figure 1A). Six of these patients had two or more urine specimens with these viral changes. UCS were obtained from 25 males with a median age of 68.6 years (range 25–90) and 5 females with a median age of 55 years (range 38–80). All clinicopathological data of the patients are summarized in Table 1. The follow-up period ranged from 7 months to 170 months, with a median of 70.5 months following or preceding the urinary cytology. All 30 patients showed typical decoy cells in their urine specimens by cytology. These cells were characterized by an enlarged nucleus with an intranuclear inclusion, which has an amorphous basophilic ground-glass appearance (Figure 1).

### 3.2. BKPyV in Patients without a History of Urothelial Cell Carcinoma

Of the thirty patients, sixteen had no history of UCC (53.3%). Out of the sixteen patients without a history of UCC, five patients (5/16; 31.25%) had a history of kidney transplantation, and one patient (1/16; 6.25%) had a history of stem cell transplantation. Other four patients (4/16; 25%) from the non-UCC history group had a history of chronic diseases that required immunosuppression and systemic therapy/radiotherapy, such as ulcerative colitis, diabetic nephropathy, autoimmune thyroiditis, autoimmune hepatitis, and not-urological cancer (prostate adenocarcinoma, colon adenocarcinoma, high-grade sarcoma of the soft tissue). The other six patients (6/16; 37.5%), who had follow-up history in the database for at least one year after the first presentation with BKPyV positive urine cytology, did not have any history of chronic diseases, nor were diagnosed with UCC during the follow-up period (Table 1 and Supplementary Table S1). Those six patients could be considered as immunocompetent for the period of their follow-up in our institution. Of the sixteen non-UCC patients, thirteen (81.25%) were positive for BKPyV IHC in urinary samples (Figure 1C). IHC could not be performed in three patients because no material was left due to multiple usages for additional analyses (Table S1).

### 3.3. Presence of BKPyV in Urothelial Cell Carcinoma

All fourteen patients with a history of UCC developed cancer prior to the detection of BKPyV-IHC positive decoy cells in their urine. Decoy cells in their urine were detected in the follow-up after the first presentation with UCC with a median period of 3.5 years (range 1–13 years) and were positive for the SV40 LTAg by immunohistochemistry, indicating the BKPyV-reactivation in urothelial cells (Figure 1C; Table 2; Supplementary Table S2). Additionally, BKPyV-DNA PCR of the five available urine sediments revealed BKPyV-positivity as confirmed by sequencing (Table 2). Interestingly, none of these patients had decoy cells in urine by preoperative cytology prior to the diagnosis of UCC.

**Figure 2:**
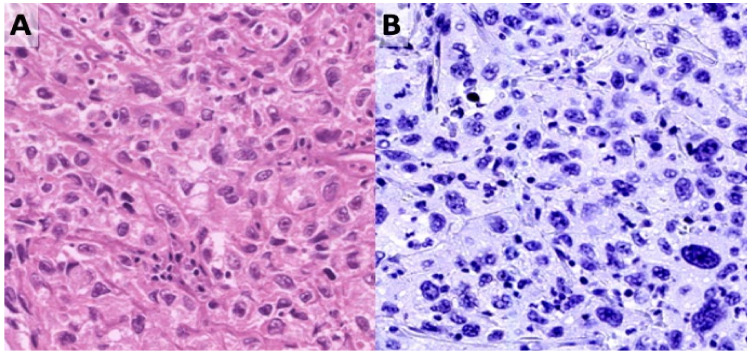
High-grade urothelial cell carcinoma and immunohistochemistry for BKPyV. (**A**) H&E-stained section shows neoplastic UCC cells with no visible viral changes in the nuclei. (**B**) a representative BKPyV-negative UCC as tested by IHC (200× magnification).

Nine of these patients (9/14; 64.3%) were known to have a history of intravesical treatment, either BCG, mitomycin, or/and radiotherapy (Table 1). The treatment history of the other five UCC patients is lacking. Two patients had recurrent UCC after intravesical treatment, and the diagnosis of BKPyV infection was confirmed by cytology. Twelve patients (12/14; 85.7%) presented with a conventional type UCC (either low-grade, high-grade UCC, or CIS) and two patients had invasive UCC with sarcomatoid differentiation (Supplementary Table S2). No micropapillary urothelial carcinoma, pleomorphic giant cell carcinoma, or bladder adenocarcinomas were found in the patients’ cohort. The H&E–tained slides of all 14 UCC patients were examined histologically and revealed no virus-related cytopathic changes (Figure 2A). BKPyV-IHC was performed on the available UCC FFPE specimens (with either in situ or invasive UCC of the urinary bladder) of 12 patients (Table 2, Supplementary Table S2). BKPyV-IHC was negative in all tested UCC samples (Figure 2B, Table 2), including metastatic and recurrent UCC. In addition, BKPyV DNA PCR was performed on the available UCC FFPE tissues of the nine patients, and all nine samples were negative for BKPyV-DNA (Table 2). Due to multiple previous histopathological and immunochemical diagnostic procedures, no FFPE tissues of the remaining five UCCs were available to perform BKPyV-IHC or PCR (Table S2).

## 4. Discussion

In this study, we evaluated the association of BKPyV-positive urine cytology with UCC and the presence of BKPyV in UCS and FFPE samples of the UCC cohort. The overall cytology results revealed a very low percentage (0.2%) of BKPyV-positive urine cytology in the database of our institution over a 15 year period (January 2004–December 2019). Several studies reported that decoy cells were detected in 18 to 28% of the urine sediments of patients with kidney transplantation [18,19,20,21,22,23]. In contrast to those studies, our study was not restricted to immunocompromised patients but also included patients with no previous history of organ transplantation, chemotherapy, radiotherapy, malignancy, or chronic disease, requiring immunosuppressive therapy (such as colitis ulcerosa, autoimmune disease, diabetes, connective tissue disease). Previous studies reported similar low detection rates of BKPyV in UCS when all patients (immunocompromised and immunocompetent) were included in the search [12,24].

The main finding of our work is that BKPyV was found in the UCS of the patients diagnosed with UCC and was detected in the follow-up period after the initial diagnosis of the UCC was made. Moreover, this BKPyV-infection may be related to the intravesical treatment of their UCC. In our study, UCC developed before the diagnosis of BKPyV-infection in urine cytology and were negative for BKPyV-IHC or PCR in FFPE tissue samples.

BKPyV has been associated with some types of UCC in the context of immunosuppression, polyomavirus-associated nephropathy (PVAN), and hemorrhagic cystitis [1,2,25,26]. The asymptomatic urinary shedding of BKPyV has been reported in healthy immunocompetent individuals as well [27]. It is known that BKPyV remains latent following the infection of the urinary tract in the proximal tubular epithelium of the kidney, the urothelial cell layer of the bladder, ureters, or renal pelvis [1]. Immunosuppression due to any clinical reason leads to the reactivation of BKPyV from viral latency [28,29]. The detection of decoy cells in voided urine is a widely used diagnostic tool as a cytopathological surrogate marker of a BKPyV-infection [30] and has several clinical implications. These cells mimic atypical neoplastic cells and can be misdiagnosed as carcinoma in situ [12]. The reactivation of BKPyV can lead to BKPyV-associated nephropathy. BKPyV-associated nephropathy develops in 1–10% of kidney transplant recipients and can ultimately result in renal graft loss in 30% up to 80% of the cases [25,28].

The prevalence of BKPyV varies from one study to another, which probably depends on the number of transplantations performed in the corresponding institution. Decoy cells are more often found in patients with a history of kidney or stem cell transplantation [25,28]. Our results show that BKPyV-positive decoy cells in voided urine samples were also detected in patients with chronic diseases, such as autoimmune diseases, diabetes, ulcerative colitis, diabetic nephropathy, and non-urological cancer, which could be explained by immunosuppressive or systemic therapy. Our findings are in line with previously reported results by Reploeg et al. [31].

Interestingly, 20% of the patients in our cohort did not have any history of immunosuppression, transplantation, or cancer, which could explain the reactivation of BKPyV. Thus, our data show that BKPyV-infection can occur in immunocompetent patients who did not have any history of UCC, organ transplantation, autoimmune or immunomodulated disease, or immunosuppressive medication. The reactivation of BKPyV was reported previously in immunocompetent patients with hemorrhagic and non-hemorrhagic cystitis, ulcerative painful bladder syndrome, and interstitial cystitis [31,32], possibly suggesting a pathophysiological role for BKPyV in the development of these diseases.

BKPyV was associated with several types of cancer, including high-grade urothelial carcinoma (both in situ and invasive), micropapillary urothelial carcinoma, pleomorphic giant cell carcinoma, bladder adenocarcinoma, and nephrogenic adenoma [3,5,6,7,8,9]. Our study did not find any of these rarely seen types of urothelial cell carcinoma. Our cohort included conventional type urothelial cell carcinoma, and none of the samples were positive for BKPyV. Similar data were published previously. Csoma et al. reported no evidence of BKPyV in 76 bladder cancer samples in their cohort [29]. Kumari et al. reported no association of BKPyV with urothelial carcinoma in immunocompetent patients [33]. Lu et al. showed no association of BKPyV cytopathic effects in urine cytology with the high risk of developing a high-grade urothelial carcinoma [24]. In our study, the BKPyV in urine was not related to the urothelial cell carcinomas resected prior to the virus detection in urine, making its role in the oncogenesis of UCC very unlikely in our cohort.

Nevertheless, based on our results, BKPyV may be associated with intravesical therapy and/or radiotherapy since nine patients with UCC (9/14:64%) in our cohort were treated with either BCG, mitomycin, or radiotherapy before the detection of the virus in urine. To the best of our knowledge, this is the first study reporting such findings. None of the patients had a decoy cell in the urine cytology prior to the diagnosis of UCC. Further research is necessary to clarify the exact mechanism of its occurrence or the nature of the association. It is known that intravesical treatment can lead to BCG cystitis and even disseminated systemic BCG infection [34,35]. Eosinophilic cystitis is a relatively common complication of intravesical mitomycin therapy [36,37]. Potentially, therapy-induced cystitis could explain the reactivation of the latent BKPyV. Still, this question was not the aim of our present study, and further research is needed to elucidate this issue. Moreover, the standard treatment of all patients diagnosed with UCC usually includes *Bacillus* Calmette–Guerin (BCG) or mitomycin C. Therefore, we cannot exclude that the other five patients with unknown UCC-treatment have also received the treatment, but we could not confirm this information in the database. However, it is known that reactivation of BKPyV can occur following various reasons, and further research is needed to elucidate its cause and mechanism in the case of intravesical treatment.

## 5. Conclusions

In our study, the BKPyV detected in urine was not associated with the prior resected urothelial cell carcinomas, potentially excluding the role of this virus in the oncogenesis of the conventional type UCC in our cohort. BKPyV-reactivation is not restricted to immunosuppression but also found in the urine of patients diagnosed with urothelial cell carcinoma and patients without any history of transplantation, malignancy, or chronic diseases. The intravesical treatment could be associated with the reactivation of the latent BKPyV. BKPyV-positivity in the immunocompetent patients may be explained by cystitis, possibly causing viral reactivation. BKPyV testing in post-UCC patients is of potential clinical relevance for the risk assessment of BKPyV-nephropathy, and further research is needed to unravel this complex relationship.

## Figures and Tables

**Table 1:** Patients clinicopathological data, demographics, and results.

Clinicopathological Characteristic	Patients (*n* = 30)
Age range (years)	25–90
Male	25–90
Female	38–80
BKPyV-IHC+ve in urine cytology	27/30
Non-UCC patients	13/16
UCC-patients	14/14
Non-UCC patients	16
Immunosuppressed	10
Transplantation	6
Renal	5
Stem cell	1
Chronic disease (IBD, AIH, CTD, other malignancy)	4
No chronic disease	6
UCC patients	14
LGUCC	6
HGUCC	1
INUCC	1
CIS	2
LGUCC and HGUCC	2
CIS and HGUCC	1
CIS and INUCC	1
UCC with treatment	9
Intravesical treatment	7
BCG	4
Mitomycin	2
Both BCG and mitomycin	1
Radiotherapy	1
Intravesical treatment and radiotherapy	1
UCC with unknown treatment	5

UCC, urothelial cell carcinoma; IHC, immunohistochemistry; +ve: positive; IBD, inflammatory bowel disease; AIH, autoimmune hepatitis; CTD, connective tissue disease; LGUCC, low-grade non-invasive urothelial cell carcinoma; HGUCC, high-grade non-invasive urothelial cell carcinoma; CIS, carcinoma in situ; INUCC, invasive urothelial cell carcinoma; BCG, *Bacillus* Calmette–Guerin.

**Table 2:** Clinical data, BKPyV IHC, and DNA PCR results.

Patient ID.	Diagnosis	(Intravesical) Treatment	BKPyV IHC		BKPyV PCR			
			Urine Cytology	FFPE Tissue	Urine Sediments		FFPE Tissue	
					LTAg	VP1	LTAg	VP1
**I.7**	LGUCC	Mitomycin	+	n.a.	n.a.	n.a.	n.a.	n.a.
**I.8**	LGUCC	Unknown	+	-	n.a.	n.a.	n.a.	n.a.
**I.9**	LGUCC	Unknown	+	-	n.a.	n.a.	n.a.	n.a.
**I.10**	LGUCC, HGUCC	BCG/Mitomycin	+	-	n.a.	n.a.	-	-
**I.12**	INUCC	Unknown	+	n.a.	n.a.	n.a.	n.a.	n.a.
**I.13**	CIS, INUCC	BCG/radiotherapy	+	-	n.a.	n.a.	-	-
**I.15**	LGUCC	Unknown	+	-	n.a.	n.a.	-	-
**I.17**	CIS	BCG	+	-	n.a.	n.a.	-	-
**I.21**	HGUCC	Radiotherapy	+	-	n.a.	n.a.	-	-
**I.22**	CIS	BCG	+	-	+	-	-	-
**I.23**	LGUCC, HGUCC	BCG	+	-	+	-	-	-
**I.26**	CIS and HGUCC	BCG	+	-	+	+	-	-
**I.29**	LGUCC	Mitomycin	+	-	+	-	n.a.	n.a.
**I.30**	LGUCC	Unknown	+	-	+	-	-	-

Patient ID, lab identification number; FFPE, formalin-fixed paraffin-embedded tissues; UCC, urothelial cell carcinomas; PCR, polymerase chain reaction; +, positive; -, negative; LTAg, large tumor antigen; VP1, viral protein; UCC, urothelial cell carcinoma; n.a., not applicable; IHC, immunohistochemistry; LGUCC, low-grade non-invasive urothelial cell carcinoma; HGUCC, high-grade non-invasive urothelial cell carcinoma; INUCC, invasive urothelial cell carcinoma; CIS, carcinoma in situ; BCG, *Bacillus* Calmette–Guerin.

## Supplementary Materials

The following are available online at https://www.mdpi.com/1999-4915/12/12/1412/s1. Table S1: Clinicopathological data, demographic and relevant follow-up information of the patients without a history of urothelial cell carcinoma. Table S2: Clinicopathological, histopathological and immunohistochemistry data in the patients diagnosed with urothelial cell carcinoma (either in situ or invasive). Table S3: PCR-primers used in this study.
