# Supplementary material for: Reactivation of BK Polyomavirus in Urine Cytology Is Not Associated with Urothelial Cell Carcinoma
Source: Viruses. 2020 Dec 8;12(12):1412. doi: 10.3390/v12121412 (PMC7763809; doi:10.3390/v12121412)
Supplement: Supplementary file 1 [file viruses-12-01412-s001.pdf]

Supplementary Materials:

Table S1. Clinicopathological data, demographic and relevant follow up information of the patients without a history of urothelial cell carcinoma.

Patient Lab-ID	Age	Gender	Follow up period (months)	First presentation with BKPyV urine cytology	BKPyV IHC on cytology	Patient history including transplantation and immunosuppression
**I.1**	25	M	12	2006	+	Kidney transplantation
**I.2**	57	F	135	2007	+	Kidney transplantation
**I.3**	50	M	134	2007	n.a.	Colitis ulcerosa
**I.4**	60	M	7	2008	+	Kidney transplantation
**I.5**	74	M	12	2008	+	Kidney transplantation
**I.6**	63	M	85	2009	+	Autoimmune thyroiditis, diabetes, prostate adenocarcinoma Grade group 1
**I.11**	67	M	12	2011	n.a.	No chronic disease
**I.14**	72	M	75	2012	+	Kidney transplantation 2011
**I.16**	55	F	82	2012	+	Autoimmune hepatitis
**I.18**	66	M	27	2014	+	2014 colon adenocarcinoma. 2015 High grade sarcoma soft tissue leg. No information about treatment.
**I.19**	78	M	12	2014	+	No chronic disease
**I.20**	38	F	22	2014	+	No chronic disease
**I.24**	77	M	51	2015	+	No chronic disease
**I.25**	69	M	12	2016	+	Stem cell transplantation, GVHD liver.
**I.27**	76	M	16	2016	n.a.	No chronic disease
**I.28**	45	F	48	2017	+	No chronic disease

Abbreviations: M, Male; F, Female; BKPyV, BK polyomavirus; n.a. no material available for immunohistochemistry.

Table S2. Clinicopathological, histopathological and immunohistochemistry data in the patients diagnosed with urothelial cell carcinoma (either in situ or invasive).

Patient Lab-ID	Age	Gender	Follow up period (months)	Presentation UCC (Year)	First presentation with BKPyV in urine cytology (Year)	Number of years between presenting UCC and detecting BKPyV urine cytology	pT stage	Patient history
**I.7**	83	M	66	2006	2010	4	pTa	- 2004: LGUCC of the distal ureter and nephrectomy.<br>- 2006: First presentation with LGUCC urinary bladder.<br>- 2008: Last presentation LGUCC urinary bladder.<br>- No tissue blocks available for IHC.
**I.8**	55	M	56	2009	2010	1	pTa	- 2009: LGUCC urinary bladder. Negative BKPyV-IHC of the UCC TURB.<br>- 2010 and 2018: BKPyV positive in urine.
**I.9**	71	M	169	2005	2010	5	pTa	- 2005: First presentation LGUCC urinary bladder.<br>- 2013: Negative BKPyV-IHC of the UCC TURB.<br>- History of prostate adenocarcinoma.
**I.10**	89	M	132	2008	2010	2	pT1 and later pTa	- 2008: HGUCC. Negative BKPyV-IHC of the UCC TURB.<br>- 2009 LGUCC. Negative BKPyV-IHC of the UCC TURB.
**I.12**	90	M	85	2004	2011	7	pT2	- pT2G3 detrusor invasive UCC with sarcomatoid features.<br>- No tissue blocks available for IHC.
**I.13**	55	M	88	2008	2011	3	pTis progressed to pT3 and multiple metastases	- 2008: CIS with BCG treatment. Negative BKPyV-IHC of CIS specimen.<br>- 2010: INUCC with radiotherapy and chemotherapy. Negative BKPyV-IHC.<br>- 2012: lymph node metastasis. Negative BKPyV-IHC.<br>-2014: bladder resection from pT3 (INUCC); on two samples of metastatic UCC in rectum. Negative BKPyV-IHC.<br>- 2015: small intestine resection with metastases UCC and lymph node metastasis. Negative BKPyV-IHC.
**I.15**	76	M	10	2011	2012	1	pTa	- 2011: LGUCC. Negative BKPyV-IHC in the TURB sample
**I.17**	74	M	94	2011	2013	2	pTis	- 2011: CIS urinary bladder. Negative BKPyV-IHC in the TURB samples.
**I.21**	66	M	75	2013	2014	1	pTa	- 2013: HGUCC in the diverticulum of the urinary bladder, with partial squamous differentiation. Negative BKPyV-IHC in the samples of high grade UCC from the diverticulum of the urinary bladder, with partial squamous differentiation;

**I.22**	70	M	115	2008	2015	7	pTis	- 2018: Negative BKPyV-IHC in the urinary bladder biopsy with chronical inflammation<br>- 2008 and 2014: Multiple CIS with multiple BCG courses. Negative BKPyV-IHC.
**I.23**	72	M	134	2007	2015	8	pT1	- 2007: INUCC. Negative BKPyV-IHC in high grade stromal invasive UCC.<br>- 2015 and 2018: Negative BKPyV-IHC of non-invasive LGUCC.
**I.26**	80	F	78	2011	2016	5	pT1, pTis	- 2011 and 2014: Multiple INUCC and CIS in the period. Negative BKPyV-IHC.<br>- 2018: Liver metastasis UCC. Negative BKPyV-IHC.
**I.29**	59	M	24	2017	2018	1	pTa	- 2017: LGUCC. Negative BKPyV-IHC in the TURB.
**I.30**	78	M	170	2005	2018	13	pTa	- 2005 and 2018: LGUCC. Negative BKPyV-IHC.

Abbreviations: UCC, Urothelial cell carcinoma; M, Male F, Female; n.a, not applicable; IHC, Immunohistochemistry; LGUCC, Low-grade non-invasive urothelial cell carcinoma; HGUCC, high-grade non-invasive urothelial cell carcinoma; INUCC, invasive urothelial cell carcinoma; CIS, Carcinoma in Situ; BCG, Bacillus Calmette-Guerin ; FFPE, Formalin-Fixed Paraffin-Embedded ; BKPyV, BK polyomavirus; pTa, non -invasive stage; pT1, invasive stage in the stroma of urinary bladder; pT2- invasive stage in the detrusor muscle of urinary bladder; pT3 – invasive stage in the fibro-adipose tissue beyond urinary bladder.

Table S3. PCR-primers used in this study.

#	Primer	Gene	Region	Forward primer (5' to 3')	Reverse primer (5' to 3')	Products Size (bp)	Reference
**1**	BKPyV TA	LTAg	(4322-4567)	ACAGCAAAGCAGGCAAG	GGTGCCAACCTATGGAACAG	246	(1)
**2**	BKPyV VP	VP1	(1600-1726)	GCAGCTCCCAAAAAGCCAAA	CTGGGTTTAGGAAGCATTCTA	127	(2)

Abbreviations: LTAg, large tumor antigen; TA, tumor antigen; VP, viral protein; bp, base pair.

1. Narayanan M, Szymanski J, Slavcheva E, Rao A, Kelly A, Jones K, et al. BK virus associated renal cell carcinoma: case presentation with optimized PCR and other diagnostic tests. Am J Transplant. 2007;7(6):1666-71.
2. Randhawa P, Kant J, Shapiro R, Tan H, Basu A, Luo C. Impact of genomic sequence variability on quantitative PCR assays for diagnosis of polyomavirus BK infection. J Clin Microbiol. 2011;49(12):4072-6.
